# Mechanical Properties Changes of Irradiated Thermoplastic Elastomer

**DOI:** 10.3390/polym10010087

**Published:** 2018-01-17

**Authors:** David Manas, Ales Mizera, Miroslav Manas, Martin Ovsik, Lenka Hylova, Stanislav Sehnalek, Pavel Stoklasek

**Affiliations:** 1 CEBIA-Tech, Faculty of Applied Informatics, Tomas Bata University in Zlin, Nad Stranemi 4511, 760 05 Zlín, Czech Republic; dmanas@utb.cz (D.M.); manas@utb.cz (M.M.); sehnalek@fai.utb.cz (S.S.); pstoklasek@utb.cz (P.S.); 2 Faculty of Technology, Tomas Bata University in Zlin, Vavreckova 275, 760 01 Zlín, Czech Republic; ovsik@utb.cz (M.O.); hylova@utb.cz (L.H.)

**Keywords:** thermoplastic elastomer, tensile test, micro-hardness testing, radiation cross-linking, mechanical properties

## Abstract

Some polymers need a cross-linking agent for the controlled cross-linking process of polymers with a tendency to degradation during the radiation cross-linking process. While, on the other hand, other polymers do not need a cross-linking agent—predominantly there are cross-linking polymers. The Thermo-Plastic Elastomer (TPE) that was used belongs to this group of predominantly cross-linking polymers; however, this agent is added because of faster reaction times and smaller irradiation doses. Microindentation–tensile and tensile impact tests were carried out on a thermoplastic sample—with, and without, a cross-linking agent. Small changes were measured between these materials at low radiation doses, (up to 66 kGy); nevertheless, at higher doses, the influence of the cross-linking agent on the mechanical properties is significant.

## 1. Introduction

In comparison to thermoplastics, rubber materials consist of long polymeric chains that have a high degree of mobility and flexibility, and are joined into a network structure. The very high deformability of rubbers is caused by the above-mentioned mobility and flexibility. Long chains may transform their configuration rapidly because of the chain mobility factor. After cross-linking, the chains transform into a network structure, the system starts to have solid-like features, where the chains are prevented from flowing into each other under external stress, which causes a typical rubber to be able to be stretched up to 10 times its original length. According to their material properties, Thermo-Plastic Elastomers (TPEs) are included among rubber and thermoplastics materials. Most thermoplastics elastomers are a phase-separated system; usually, one phase is solid and hard at ambient temperature, while the other is in the form of an elastomer. The phases are bonded chemically by graft polymerization or block polymerization. The hard phase causes the strength of TPEs and represents the physical cross-links. The elastomer phase causes the elasticity and flexibility of the system. After melting the hard phase—or its dissolution in a solvent—the TPE can flow and can be processed by normally used processing methods and, after cooling or evaporation of the solvent, the hard phase becomes solid again and the material regains its elasticity and strength back [1].

One way that TPEs’ properties can be modified is by means of electron-beam radiation cross-linking that can cause effective improvements in this material. Generally, it is a well-known fact that some polymers like natural rubber and ethylene propylene rubber can be cross-linked by electron-beam radiation, while some other polymers—like polypropylene and poly(vinyl chloride)—have a tendency to degrade. Some polymers are able to cross-link on their own, while some need to be formulated with a cross-linking agent, and modification is used during their polymerization process. Ditrimethylol propane tetraacrylate, ethylene-vinyl acetate (EVA) and low-density polyethylene were blended in varying proportions and compression moulded into the form of 0.025 ± 0.003 mm thick sheets. These sheets were cross-linked by electron-beam radiation and then subjected to many measurements. It was discovered that the crystalline phase of the blends is affected by electron-beam radiation only at higher doses (200 kGy and above), the gel fraction increases with increases of the radiation dose, and the ethylene-vinyl acetate and ditrimethylol propane tetraacrylate content. The morphology of blends does not change after irradiation [2,3].

It is also possible to stabilize foam formations by irradiation cross-linking. This also depends on the chemical blowing agent and the dose of irradiation that affects the density of the foam; thus, it is possible to create Thermo-Plastic Natural Rubber (TPNR) foams, which were studied by scientists from Malaysia and the U.K [4] who cooperated in this study. They used uncured natural rubber and EVA blends (60:40) with various chemical blowing agents, and these blends were compression-moulded in the form of plaques and subsequently cross-linked by doses of 30, 40, 60, 80, 100, 120, 150 and 200 kGy [4].

Scientists from Tomas Bata University in Zlin [5] studied the influence of electron-beam radiation on the micro-mechanical properties of TPE. They found that—because of the chemical composition changes of TPE after irradiation by electron-beam radiation— indentation hardness, indentation elastic modulus, and indentation creep decreased slightly with the increasing radiation dose, which caused the worsening of the micro-mechanical properties of this material. In this case, it is very important to determine the appropriate radiation dose needed for each material to lead to improvements in material properties [5]. Kieran A. Murray, James E. Kennedy, Brian McEvoy, Olivier Vrain, Damien Ryan, Richard Cowman and Clement L. Higginbotham [6] investigated different ways of applying TPE irradiation and the mechanical, thermal, structural and physicochemical behaviours of TPE after irradiation. They measured that the higher the irradiation dose, the lower the tensile strength and also the lower elongation at break-point, while the higher the dose of irradiation, the higher the Young’s modulus of TPE and also Shore D hardness were. The melt-flow index of TPE decreases with the radiation dose [6].

Many other research papers have concentrated on the chemical structure and mechanical properties of TPE blended from different materials—with or without cross-linking (electron-beam, chemical or gamma radiation) and the material’s behaviour before and after cross-linking. This depends on the application and on the products. In the case of foams, cross-linking is desirable for cells in foam stabilization. In the case of required higher elasticity, cross-linking is not the best way to modify TPE [7,8,9,10,11].

The effect of the electron-beam radiation dose and temperature on aliphatic thermoplastic polyurethane was studied by Adem, Angulo-Cervera, et al. [12]. As a material, they used aliphatic polyurethane—which they subsequently irradiated by doses ranging from 300 to 4000 kGy, in air, at 100 ${}^{\circ }$C. The consequence of such cross-linking was a lowering of stress and strain at break levels, while Young’s Modulus was without change-up the maximum dose of 4000 kGy, where it increased significantly. The irradiation temperature did not cause a great effect on mechanical properties—up to the dose of 4000 kGy, where Modulus significantly increased [12].

Poly(aliphatic/aromatic-ester) (PED) containing poly(butylene terephthalate) sequences extended with the butylene ester of dimerized fatty acid was studied after irradiation by electron-beam radiation at the doses of 50, 75 and 100 kGy at room temperature. After irradiation, PED contained a higher gel content, which led to a higher Young’s modulus caused by cross-linking the material. Cross-linking also caused a slight increase of the melting temperature and the decrease of crystallinity [13]. The blend irradiated by electron-beam radiation at doses ranging from 50 to 250 kGy, at ambient temperature, containing ethylene vinyl acetate (EVA) 18 % from Hyundai, (Seoul, South Korea), and thermoplastic polyurethane (TPU) from Bayer AG (Leverkusen, Germany) were investigated. From the mechanical properties’ point of view, the modulus increased along with the increasing radiation dose, while tensile strength and elongation at break showed drops with the increasing radiation dose [14].

It is also possible to modify blends by fillers and subsequently to irradiate the whole system. This is the case of scientists from India, namely Hui, Mushtaq, Chaki, and Chattopadhyay [15], who investigated low-density polyethylene and EVA copolymer blends containing 40 % vinyl acetate. As a filler, they used silicon dioxide nanoparticles (with particle sizes of 10–15 nm), at concentrations of 1.5, 3 and 5 wt %, and they irradiated these blends in air at an ambient temperature with doses of 20 and 40 kGy. After comparison of non-irradiated filled systems and irradiated filled systems of this type, the result was the increase in elastic modulus and melt viscosity after irradiation [15].

Electron-beam radiation is also able to improve some recycled blends properties—like Low Density Polyethylene/Ethylene-Vinyl Acetate (LDPE/EVA) ground tyre rubber (GTR) blends, this was the aim of Hungarian scientists [16]. They created thermoplastic elastomer blends with different concentrations of EVA content containing recycled LDPE (40 wt %) and LDPE + EVA copolymer (30 wt %)—as the EVA content rises, the LDPE ratio simultaneously decreases, and the GTR (30 wt %). Their cyclic tests showed increases in total deformation associated with increasing EVA content. The tensile strength of irradiated blends decreased with higher content of EVA in comparison with non-irradiated blends, while, on the contrary, elongation at break increased with the increasing content of EVA in both cases of non-irradiated and irradiated blends in a similar manner [16].

The main aim of this study is to compare changes in the mechanical properties of irradiated thermoplastic elastomers, with and without a cross-linking agent. A cross-linking agent usually helps to foster faster reactions in materials and also small irradiation doses can be used for particular improvement of the properties. The use of a cross-linking agent or not can be influenced by many internal or external reasons, and an end-product with a cross-linking agent may not work as it would work without it, and vice versa.

## 2. Materials and Methods

### 2.1. Material Preparation

**Figure 1:**
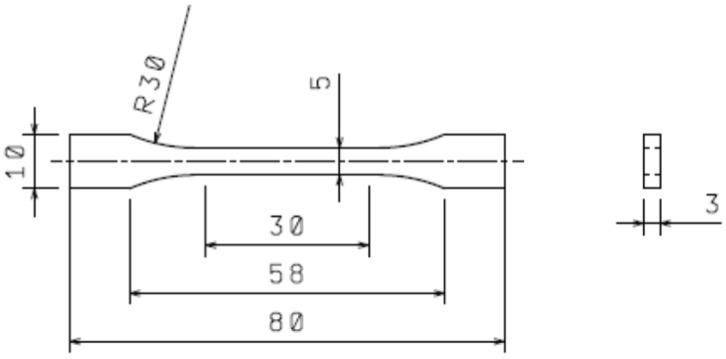
Dimensions of testing specimen.

A thermoplastic elastomer without (TPE, PTS-UNIFLEX-E25D/M*M800 natural) and with (V-TPE, V-PTS-UNIFLEX-E25D/M*M800/20 natural) a cross-linking agent were used as the basic polymer materials. The general information on these materials is depicted in Table 1. An ARBURG Allrounder 170U 150-30 injection-moulding machine (Loßburg, Germany) was used for sample preparation, with the processing conditions set to comply with the TPE producer’s recommendations (Table 2). 1BA type samples were used for all measurements—which were produced according to the ISO 527-2 standard. The dimensions and shape of the test bodies is apparent from Figure 1.

Irradiation of the tested TPE was performed with the kind help of BGS Germany (Wiehl, Germany), in the BGS Wiehl plant, using accelerated electrons. A Rhodotron*®* E-beam accelerator (Tongeren, Belgium) with 10 MeV electron energy was used for this purpose. The irradiation process of the TPE specimens was performed under general conditions, (air atmosphere, ambient temperature 23 ${}^{\circ }$C) just as it is done in engineering practice. The range of the doses was determined on the basis of experience gained from industrial irradiation practice, in the range of 33 to 198 kGy. Each passage under the accelerator scanner is equal to 33 kGy. The required dose was determined according to accelerator parameters and its correctness was measured by a dosimeter. A Nylon FWT 60-00 dosimeter (Goleta, CA, USA) was used to check the correct radiation dose, followed by analysis carried out on a Genesys 5 spectrophotometer (Madison, WI, USA)—in line with the American Society for Testing and Materials - ASTM 51261 standard. The required and real surface values of irradiation dose gains are depicted in Table 3.

### 2.2. Gel Content

A gel (content) test is performed in order to determine the non-dissolved gel content of the given material—according to the ASTM D 2765 standard—Test Method C. A portion of 0.5 g (of electron-beam irradiated TPE and V-TPE material) weighed with a precision of five decimal places on a “SWISS MADE EP 125 SM” weighing apparatus (Dietikon, Switzerland) was mixed with 100 mL of solvent. Xylene was used on the TPE because it dissolves the amorphous part of this material, and the cross-linking part does not dissolve. The mixture was extracted for 24 h. Then, the solutes were separated by distillation. After removing the residual xylene, the cross-linked extract was dried for 8 h, in a vacuum, at 100 ${}^{\circ }$C. The dried and cooled residue was weighed again with a precision of five decimal places and compared to the original weight of the portion. The result is stated in percentage as the degree of cross-linking:

(1) $$G_{i}\;=\;\frac{m_{3}-m_{1}}{m_{2}-m_{1}}\;100,$$

where $G_{i}$ is the degree of cross-linking of each specimen expressed in percentage, $m_{1}$ is the weight of the cage and lid in milligrams, $m_{2}$ is the total of the weights of the original specimen, cage and lid in milligrams, and $m_{3}$ is the total of the weights of the residue specimen, cage and lid in milligrams.

### 2.3. Micro-Indentation

Micro-indentation test was performed using a Micro-indentation tester (Micro Combi Tester), Anton Paar (Graz, Austria) according to the ISO 14577 standard. The Depth Sensing Indentation (DSI) method used enables measuring force acting on the Vickers indentor (Anton Paar, Graz, Austria), which is made of diamond (the shape of a cube corner) and the displacement of the indentor during the test. In the present study, the maximum load used was 1 N and loading and unloading rate was 2 N min${}^{-1}$. A holding time was 90 s.

**Figure 2:**
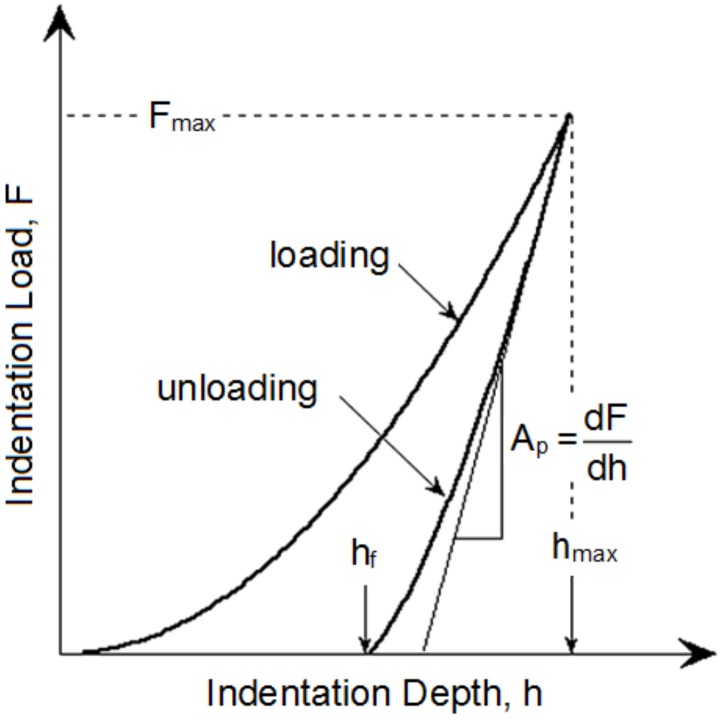
Schematic illustration of indentation curve [17].

The indentation hardness ($H_{IT}$) was calculated as maximum load ($F_{max}$) to the projected area of the hardness impression ($A_{p}$) and the indentation modulus ($E_{IT}$) is calculated from the Plane Strain modulus ($E^{*}$) using an estimated sample Poisson’s ratio (υ) according to (Figure 2) [17,18]:

(2) $$H_{IT}=\frac{F_{max}}{A_{p}},$$

(3) $$E_{IT}=E^{*}\left(1-\upsilon s^{2}\right).$$

**Figure 3:**
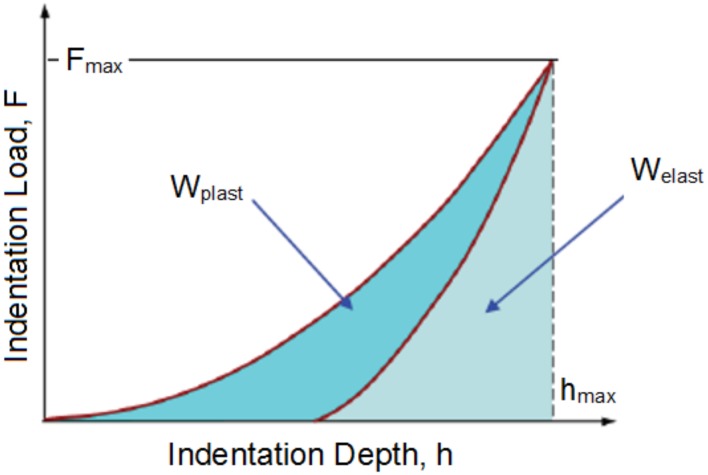
Indentation work $\eta_{IT}$.

Indentation work (Figure 3) [17,18]:

(4) $$\eta_{IT}=\frac{W_{elast}}{W_{total}}100,$$

(5) $$W_{total}=W_{elast}+W_{plast}.$$

Measurements of all above-mentioned properties were performed 30 times to ensure statistical correctness.

### 2.4. Tensile Test

The tensile test was carried out on a T 2000 Alpha Technologies testing machine (Hudson, OH, USA) at ambient temperature 23 ${}^{\circ }$C according to ISO 37 standard, under a constant speed of elongation of 500 mm min${}^{-1}$. The used testing samples were in the shape of a shovel, as it displayed in Figure 1. They tested 15 samples and their values of tensile strength, elongation and modulus 50, 100, 200, 300 and 500 were evaluated in programs TestXpert II (V3.31, Zwick/Roell, Ulm, Germany), MS Excel (2016, Microsoft, Redmond, WA, USA) and MiniTab (v16, State College, PA, USA). In all figures, arithmetic mean and standard deviation are used.

### 2.5. Impact Tensile Test

The tensile impact test was carried out on Zwick HIT50P equipment (Ulm, Germany) at ambient temperature 23 ${}^{\circ }$C according to standard ISO 8256. In this test impact hammer, 50 J of potential energy was used. There were tested 15 samples (Figure 1) and their values of maximum impact force and elongation were evaluated in programs TestXpert II, MS Excel and MiniTab. In this measurement, arithmetic mean and standard deviation were also used as the statistical parameters.

## 3. Results and Discussion

### 3.1. Gel Content Determination

**Figure 4:**
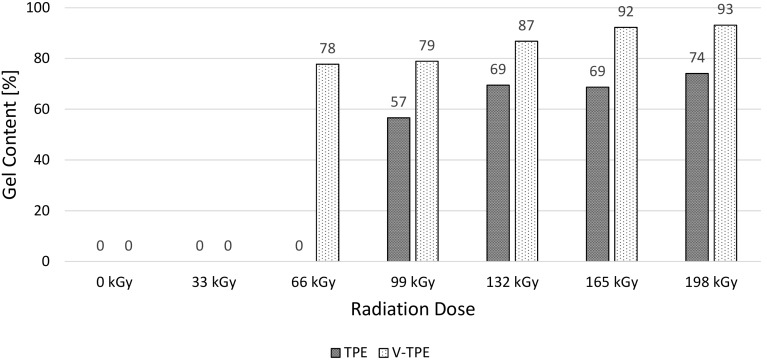
Gel content of irradiated TPE and V-TPE.

Gel content of tested specimens was measured extraction in xylene according to ASTM D 2765—test method C. In Figure 4, gel content of TPE and V-TPE is displayed. From the figure, the influence of a cross-linking agent is possible to observe. TPE without a cross-linking agent has lower gel content than V-TPE with a cross-linking agent. TPE at doses 0, 33 and 66 kGy, and also V-TPE at doses 0 and 33 kGy, were dissolved. TPE is cross-linkable by electron beam radiation without a cross-linking agent; however, a dose of irradiation must be higher to achieve relevant changes of properties and also the value of gel content is lower in comparison with the V-TPE—for example, at the maximum used dose of irradiation 198 kGy, gel content for TPE is 74% and for V-TPE is 93%.

### 3.2. Micro-Indentation Test

The micro-mechanical properties (i.e., indentation hardness, indentation modulus, elastic deformation work and plastic deformation work) of the surface layer were measured using the DSI method. TPE without cross-linking agent (TPE), and TPE with the cross-linking agent (V-TPE), were selected as the test materials.

**Figure 5:**
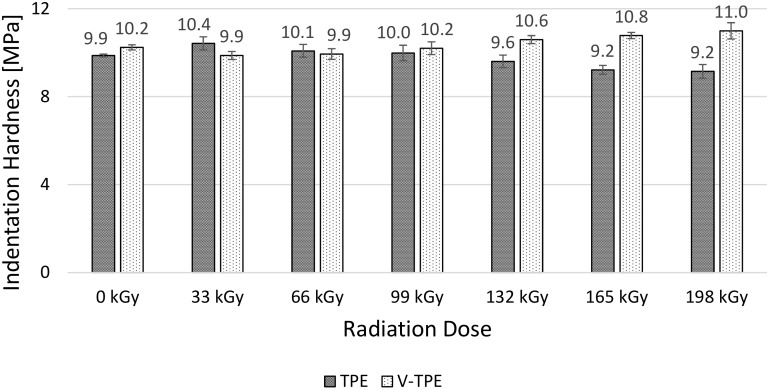
Micro-indentation hardness—TPE, V-TPE.

The micro-hardness of a TPE thermoplastic elastomer without a cross-linking agent stays approximately at the same level in the course of irradiation doses from 0 and up to 99 kGy (Figure 5). The differences in the measured micro-hardness values are in the region of the standard deviation. It is possible to note the same tendency in the case of a V-TPE thermoplastic elastomer with a cross-linking agent content irradiated by doses up to 99 kGy. Using higher irradiation doses, the micro-hardness of the TPE decreases slightly, with the lowest value occurring at the dose of 198 kGy. The micro-hardness drop reaches about 7%. On the other hand, the micro-hardness of the V-TPE rises with the same irradiation dose. The maximum micro-hardness value is measured at the dose of 198 kGy, and the difference between V-TPE and TPE is nearly 20% at the highest irradiation dose.

**Figure 6:**
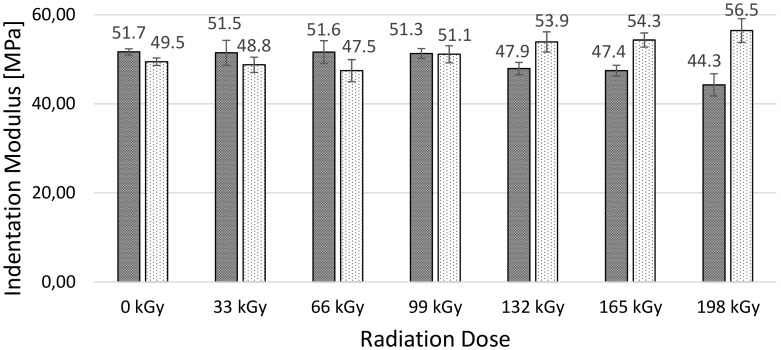
Micro-indentation modulus—TPE, V-TPE.

Such a tendency is observable when studying the indentation modulus (Figure 6). The measured values of the TPE indentation modulus continually decrease—with a maximum drop of nearly 17% at the irradiation dose of 198 kGy, while the V-TPE indentation modulus continually increases with the maximum difference of 14% at the dose of 198 kGy. From this, one can say that the difference of indentation modulus results of both TPEs studied at the 198 kGy irradiation dose reaches nearly 28%.

**Figure 7:**
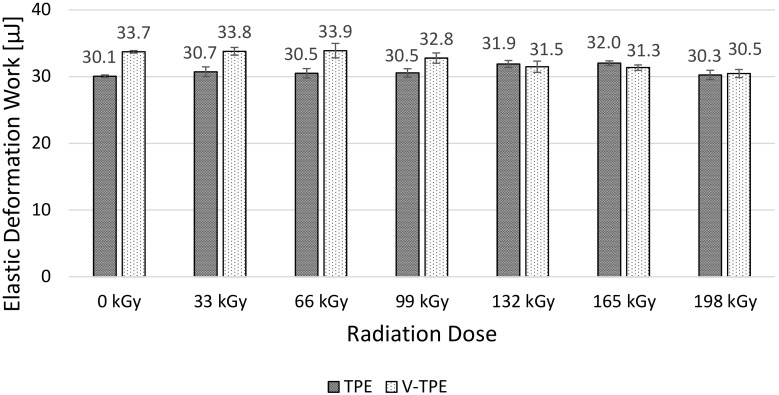
Elastic deformation work—TPE, V-TPE.

**Figure 8:**
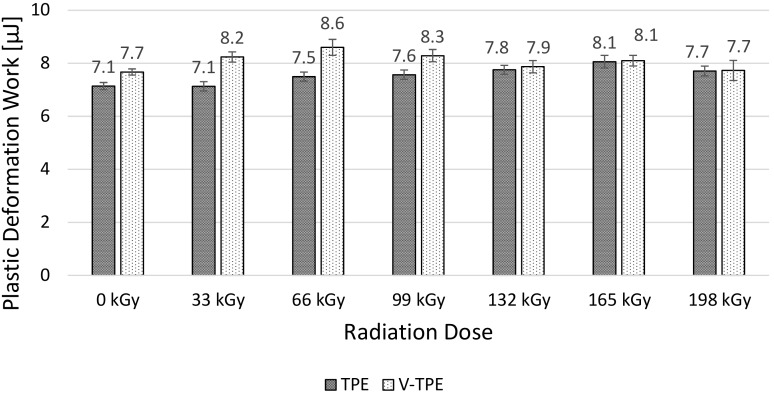
Plastic deformation work—TPE, V-TPE.

Elastic deformation work remains without significant changes (Figure 7). These values are within the standard deviation limits. Only slight changes of plastic deformation work (Figure 8) are observable in line with the increase of irradiation doses. The highest difference was measured at the range of 66 to 99 kGy.

When looking at the results of elastic (Figure 7) and plastic (Figure 8) deformation work, it is clear that, with increasing radiation dose, the TPE increase of both works was determined. On the other hand, for V-TPE, the decrease of elastic and plastic deformation work was measured. Both tested materials showed higher elastic work values in comparison with plastic deformation work values. The elastic work value was approximately four times higher as compared to plastic deformation work.

As is visible from the results of the micro-mechanical properties of the surface layer (shown) in Figure 5, Figure 6, Figure 7 and Figure 8, TPE without a cross-linking agent showed a drop in values with increasing radiation doses, which was caused by the material degradation influenced by electron beam radiation. Conversely, after addition of the cross-linking agent, the formation of a 3D net occurred and this caused the improvement of the micro-mechanical properties.

### 3.3. Tensile Test

**Figure 9:**
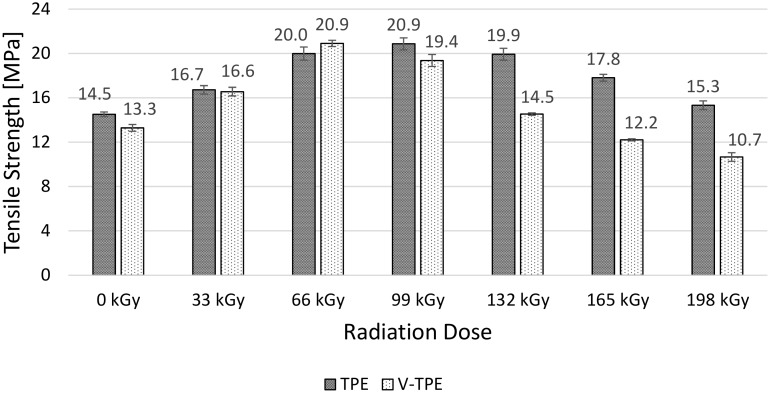
Tensile Strength—TPE, V-TPE.

After the surface properties testing procedure, tensile tests were performed on irradiated thermoplastic samples—without (TPE) and with a (V-TPE) cross-linking agent. From these measurements, it is possible to evaluate the tested material from the viewpoint of its uniaxial tensile stress, which is the basic loading of engineering parts in every application. Tensile strength shows significant changes with the irradiation dose for both TPE and V-TPE, (Figure 9). From the (non-irradiated polymers) starting point, the tensile strength increases both for the TPE and V-TPE samples, with the maximum dose of 99 kGy attained in the TPE case (a difference of 47% in comparison with non-irradiated polymers), and the dose 66 kGy in the case of V-TPE (a difference of 57% in comparison with the non-irradiated polymers). With the highest irradiation dose, both TPE and V-TPE degrade, with a significant drop in tensile strength. In the case of V-TPE, the measured tensile strength value is nearly 24% lower in comparison with the virgin polymer, and, in the case of TPE, the tensile strength value reaches the same level as those by non-irradiated ones.

**Figure 10:**
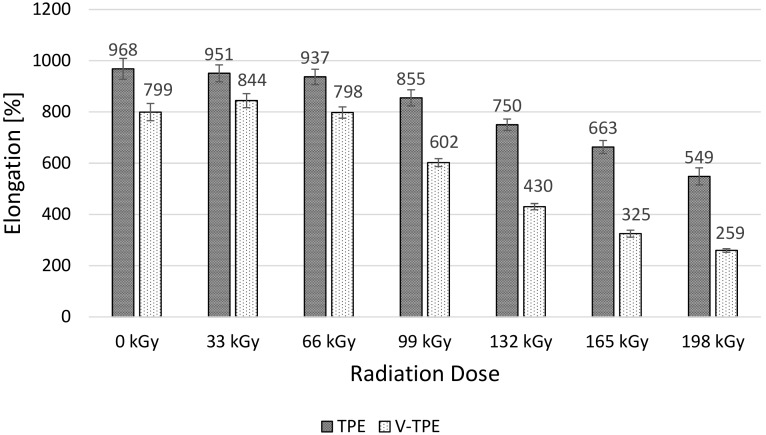
Elongation—TPE, V-TPE.

Elongation of both tested polymers goes down significantly with the irradiation dose (Figure 10). The maximum drop is measured at the irradiation dose of 198 kGy, which reaches 76% in the case of TPE—and more than twice that in the V-TPE case. It is interesting to remark that only the addition of a cross-linking agent to virgin V-TPE affects a drop in elongation of 21%, in comparison with the virgin TPE.

**Figure 11:**
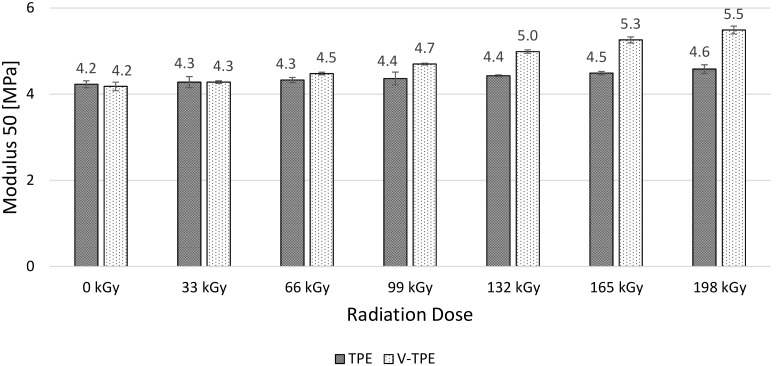
Modulus 50—TPE, V-TPE.

**Figure 12:**
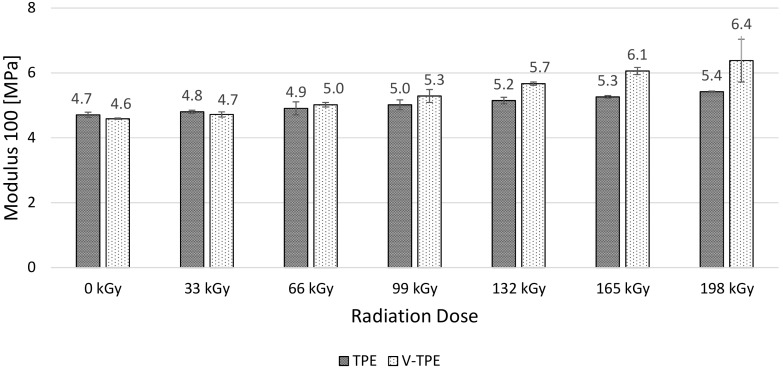
Modulus 100—TPE, V-TPE.

**Figure 13:**
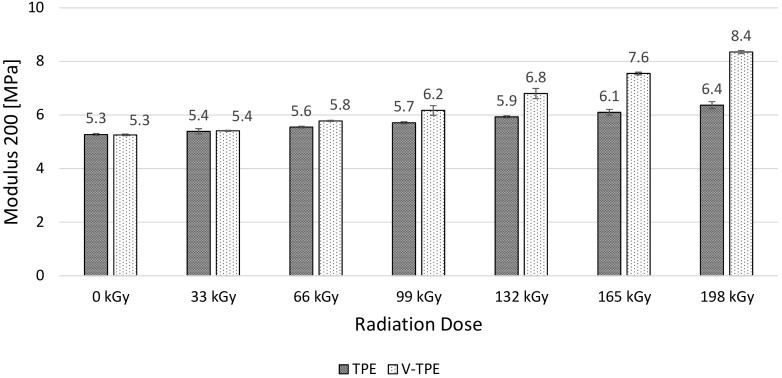
Modulus 200—TPE, V-TPE.

**Figure 14:**
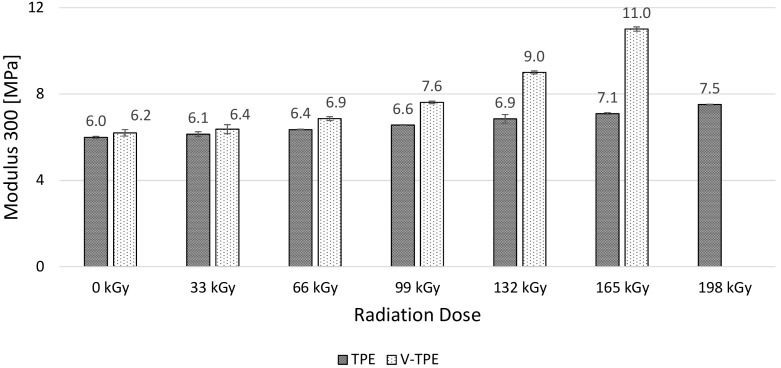
Modulus 300—TPE, V-TPE.

**Figure 15:**
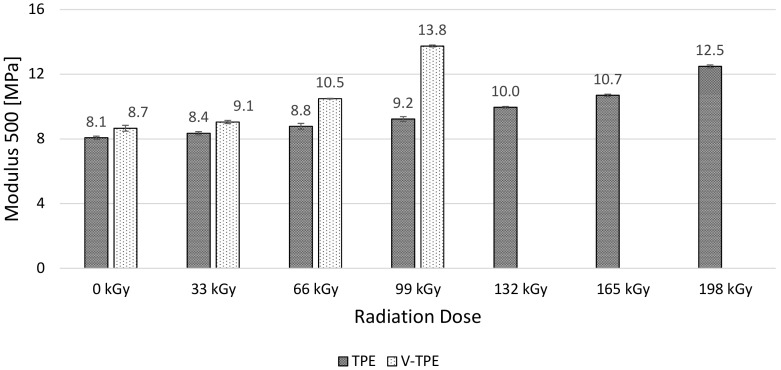
Modulus 500—TPE-E, V-TPE-E.

Modulus 50 (Figure 11), Modulus 100 (Figure 12), and Modulus 200 (Figure 13) show similar tendencies—they increase with the irradiation dose, with the highest values at the dose of 198 kGy. The increase of Modulus in the case of TPE is relatively low, lying between 10% (Modulus 50) to 20% (Modulus 200), while the V-TPE Modulus increase is more significant as compared to TPE and lies between 31% (Modulus 50) and 58% (Modulus 200). A similar tendency is shown in measurements of Modulus 300 and Modulus 500 (Figure 14 and Figure 15). Both Modulus 300 and Modulus 500 increase with the irradiation dose. Modulus 300 was not measured for V-TPE with an irradiation dose of 198 kGy because the sample cracked before this point—this is related to elongation. The same results are displayed in Figure 15, where modulus 500 is recorded up to 99 kGy for V-TPE. From these measurements, it can be seen that TPE without a cross-linking agent is more stable than with a cross-linking agent from the modulus point of view.

Determining the correlation between micro-indentation and tensile tests is very complicated due to the fact that micro-indentation tests primarily evaluate the surface layer. On the other hand, tensile tests evaluate the macro-mechanical behaviour in whole volumes. From this, it follows that the data measured from these two methods can evince different trends during the testing of irradiated materials. In this study, one can observe (in many figures) how the properties change after irradiation of TPE—with and without a cross-linking agent.

### 3.4. Tensile Impact Test

**Figure 16:**
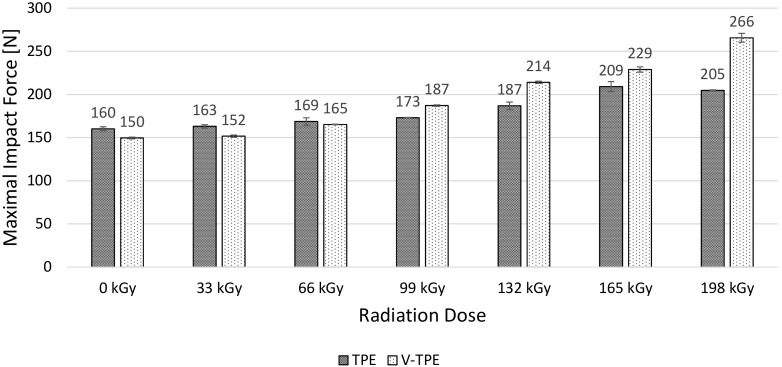
Maximal impact force—TPE, V-TPE.

**Figure 17:**
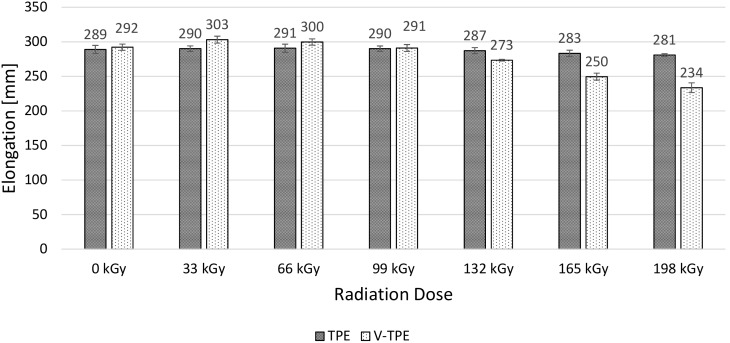
Tensile impact elongation—TPE, V-TPE.

The next test to be used was the tensile impact test with a pendulum energy of 50 J. At this set energy level, no samples were cracked, the material was simply extended and the pendulum stopped. The maximum force and elongation are displayed in Figure 16 and Figure 17, respectively. The maximal impact force change differences are about 7% between TPE and V-TPE at irradiation doses up to 66 kGy. With increasing irradiation dose, the maximal impact force rises for both materials—TPE by about 22%, and V-TPE by about 44% at maximal irradiation dose of 198 kGy, in comparison with the non-irradiated one. On the other hand, TPE elongation is almost constant through all doses of the irradiation scale. V-TPE elongation is constant up to 99 kGy, and then decreases with increasing irradiation doses; the decrease of irradiated V-TPE with 198 kGy is about 19% in comparison with the non-irradiated one. The correlation between elongation in tensile tests and impact tensile tests can be observed.

## 4. Conclusions

The thermoplastic elastomer used in this study can be cross-linked without the need for a cross-linking agent by accelerated electrons; however, cross-linking agents are used abundantly for the acceleration of the cross-linking process in working practice. From gel content measurements, it is possible to observe the huge influence of a cross-linking agent on electron-beam irradiated TPE. At the irradiation dose of 99 kGy, the TPE gel content is 57%; and 79% was measured for V-TPE. Moreover, it was found that only the addition of a cross-linking agent can cause changes in mechanical properties. For this reason, the exact material has to be chosen in final applications.

The micro-mechanical and mechanical properties of thermoplastic elastomers without (TPE) and with (V-TPE) a cross-linking agent were studied in this work. The changes in mechanical properties of thermoplastic elastomers, up to an irradiation dose 66 kGy, are similar for both materials. Significant changes in their mechanical properties occur at higher irradiation doses, which can cause the beginning of chain scissors—i.e., degradation due to irradiation. Higher doses have a greater influence on V-TPE than on TPE—that smaller doses to the cross-linking agent can cause the very rapid start of the cross-linking process and, at higher doses, cause degradation in the structure.

With increasing irradiation doses up to 66 kGy, the tensile strength rises for both materials and with similar values. With increases in the irradiation dose, the tensile strength drops; the V-TPE tensile strength at 198 kGy is lower than the non-irradiated one. The same trend was recorded at elongation of both tested materials. With each radiation dose, the elongation of V-TPE is lower than that of TPE samples. V-TPE elongation at 66 kGy is lower by about 15%, in comparison with TPE at the same radiation dose. This trend is confirmed by tensile impact tests that were performed with a pendulum energy of 50 J.

The aim of this work was to study the influence that a cross-linking agent has on thermoplastic elastomer mechanical behaviour. The sum of the results showed that a cross-linking agent has insignificant influence in the course of small irradiation doses; however, at higher doses, the influence of a cross-linking agent is huge, which correlates with gel content measurements.

## Figures and Tables

**Table 1:** TPE general information.

Properties	Test method	Unit	TPE value	V-TPE value
Hardness	ISO 868	ShA	84	87
Hardness	ISO 868	ShD	27	-
Density	ISO 1183	g cm${}^{-3}$	1.1	1.1
Tensile strength	ISO 527-1/-2	MPa	16	17.8
Elongation at break	ISO 527-1/-2	%	800	500
Melting point	ISO 11357-1/-3	${}^{\circ }$C	165	165

**Table 2:** Injection moulding parameters.

Arburg Allrounder 170U 150-30		
Injection Velocity	50	mm s${}^{-1}$
Injection Pressure	45	MPa
Cooling Time	15	s
Mould Temperature	30	${}^{\circ }$C
Holding Pressure	40	MPa
Temperature of Plasticizing Unit Zones		
Temperature under the Hopper	30	${}^{\circ }$C
Temperature Zone 1	170	${}^{\circ }$C
Temperature Zone 2	185	${}^{\circ }$C
Temperature Zone 3	200	${}^{\circ }$C
Temperature Zone 4	210	${}^{\circ }$C

**Table 3:** Irradiation dose of TPE.

Required irradiation dose (kGy)	Real surface irradiation dose (kGy)
0	0.0
33	38.7
66	77.4
99	116.1
132	154.8
165	193.5
198	232.2
